# Unraveling the Transport Properties of RONS across Nitro-Oxidized Membranes

**DOI:** 10.3390/biom13071043

**Published:** 2023-06-27

**Authors:** Davronjon Abduvokhidov, Maksudbek Yusupov, Aamir Shahzad, Pankaj Attri, Masaharu Shiratani, Maria C. Oliveira, Jamoliddin Razzokov

**Affiliations:** 1 Institute of Fundamental and Applied Research, National Research University TIIAME, Kori Niyoziy 39, Tashkent 100000, Uzbekistan; jrazzokov@gmail.com; 2 Department of Information Technologies, Tashkent International University of Education, Imom Bukhoriy 6, Tashkent 100207, Uzbekistan; 3 Institute of Material Sciences, Academy of Sciences, Chingiz Aytmatov 2b, Tashkent 100084, Uzbekistan; 4 R&D Center, New Uzbekistan University, Mustaqillik Avenue 54, Tashkent 100007, Uzbekistan; maksudbek.yusupov@uantwerpen.be; 5 Department of Power Supply and Renewable Energy Sources, National Research University TIIAME, Kori Niyoziy 39, Tashkent 100000, Uzbekistan; 6 Laboratory of Thermal Physics of Multiphase Systems, Arifov Institute of Ion-Plasma and Laser Technologies, Academy of Sciences of Uzbekistan, Tashkent 100125, Uzbekistan; 7 Research Group PLASMANT, Department of Chemistry, University of Antwerp, Universiteitsplein 1, 2610 Antwerp, Belgium; mariacecilia.oliveira@uantwerpen.be; 8 Modeling and Simulation Laboratory, Department of Physics, Government College University Faisalabad (GCUF), Allama Iqbal Road, Faisalabad 38040, Pakistan; aamir.awan@gcuf.edu.pk; 9 Center of Plasma Nano-Interface Engineering, Kyushu University, Fukuoka 819-0395, Japan; chem.pankaj@gmail.com (P.A.); siratani@ed.kyushu-u.ac.jp (M.S.); 10 Faculty of Information Science and Electrical Engineering, Kyushu University, Fukuoka 819-0395, Japan; 11 School of Engineering, Akfa University, Milliy Bog Street 264, Tashkent 111221, Uzbekistan

**Keywords:** cold atmospheric plasma, reactive oxygen and nitrogen species, nitro-oxidized membranes, molecular dynamics, free energy profile

## Abstract

The potential of cold atmospheric plasma (CAP) in biomedical applications has received significant interest, due to its ability to generate reactive oxygen and nitrogen species (RONS). Upon exposure to living cells, CAP triggers alterations in various cellular components, such as the cell membrane. However, the permeation of RONS across nitrated and oxidized membranes remains understudied. To address this gap, we conducted molecular dynamics simulations, to investigate the permeation capabilities of RONS across modified cell membranes. This computational study investigated the translocation processes of less hydrophilic and hydrophilic RONS across the phospholipid bilayer (PLB), with various degrees of oxidation and nitration, and elucidated the impact of RONS on PLB permeability. The simulation results showed that less hydrophilic species, i.e., NO, NO_2_, N_2_O_4_, and O_3_, have a higher penetration ability through nitro-oxidized PLB compared to hydrophilic RONS, i.e., HNO_3_, *s-cis-*HONO, *s-trans-*HONO, H_2_O_2_, HO_2_, and OH. In particular, nitro-oxidation of PLB, induced by, e.g., cold atmospheric plasma, has minimal impact on the penetration of free energy barriers of less hydrophilic species, while it lowers these barriers for hydrophilic RONS, thereby enhancing their translocation across nitro-oxidized PLB. This research contributes to a better understanding of the translocation abilities of RONS in the field of plasma biomedical applications and highlights the need for further analysis of their role in intracellular signaling pathways.

## 1. Introduction

A cold atmospheric plasma (CAP) is a non-equilibrium plasma operating at atmospheric pressure and room temperature [1,2,3]. It is generated by applying a high-voltage and high-frequency electrical discharge to gases such as helium, argon, nitrogen, oxygen, or air [4,5]. CAPs are increasingly being investigated for their potential applications in various fields, including biomedicine, agriculture, and materials science, due to their ability to generate a variety of reactive species, such as reactive oxygen species (ROS), reactive nitrogen species (RNS), or both reactive oxygen and nitrogen species (RONS) [6,7]. These species have been shown to have beneficial effects in biomedical applications, including cancer treatment [8] and wound healing [9]; in material processing, such as surface modification [10]; as well as in the sterilization and decontamination of bacteria, fungi, and viruses [11,12,13]. Understanding the underlying mechanisms, especially the interaction of CAP with biological systems is an active area of research, with the potential for many practical applications.

The ability of CAPs to generate reactive species (ROS, RNS, and RONS) has been shown to be beneficial in a variety of applications [14,15,16]. For instance, CAPs have been probed for cancer treatment because of their capability to selectively induce apoptosis in cancer cells, causing oxidative damage to cellular components [17]. In addition, CAPs have manifested auspicious outcomes in the context of wound healing, owing to their potential to stimulate growth factor production and improve blood circulation [9,18]. CAP-generated RONS possess the ability to amend the surface characteristics of exposed matter by disrupting and producing chemical bonds, resulting in variations to the surface morphology and chemical composition [19]. Oxidative damage to lipids is of particular significance to our study, as lipids form the bulk of the phospholipid membrane. Damage to lipid molecules alters the membrane’s properties, which in turn affects its permeability.

The generated during CAP treatment initially interact with the water on the cell surface, before affecting the cell membrane [20]. Subsequently, RONS penetrate into the cell, inducing oxidative stress in the membrane, thereby forming lipid peroxide as the primary oxidation product and aldehyde, alcohol, and ketone as secondary products [21,22,23]. Both experiments and simulations have been used to study the effect of lipid and cholesterol oxidation on membrane properties [24,25,26,27,28,29]. Cholesterol is crucial for maintaining the fluidity and stability of cell membranes, preventing phase transitions in lipids that can disrupt cellular processes [30]. These investigations have shown that oxidized lipids and cholesterol change the structure and dynamics of the membrane. As a result, the molecular characteristics of the membrane are altered, leading to increased permeability and fluidity, while decreasing the lipid order and bilayer thickness [31,32,33]. Moreover, the interaction of RNS with the membrane leads to the formation of various nitration products, primarily nitro phospholipids [34]. This process, similarly to lipid oxidation, is considered a significant source of nitrated lipids, resulting in high yields. The modeling results revealed that due to the impact of nitration, the water permeability of nitrated membranes increased three times compared to oxidized membranes [35]. Numerous investigations have been accomplished utilizing computer simulations, to examine the transfer of reactive species through biological membranes [32,36,37]. These studies indicated that plasma-generated reactive species can penetrate through the cell membrane and interact with the membrane lipids and transmembrane proteins [38,39]. Additionally, aquaporins can act as transport channels for reactive species across the membrane, and plasma treatment can induce changes in membrane properties that enhance the permeation of reactive species [32]. However, the specific transport mechanisms and the effects of different reactive species on membrane permeability require further investigations.

The above studies investigated the permeation of RONS across oxidized, and to some extent through nitrated, membranes. However, the permeation of RONS through both oxy-nitrated membranes has not yet been investigated. It is expected that the combination of oxidative and nitrative damage could have an additive or synergistic effect on membrane properties, leading to more significant changes in the permeability and stability of the membrane. Therefore, further studies are needed to elucidate the synergistic effect of oxy-nitration on membrane properties. These findings could significantly contribute to a better understanding of the effect of CAP in plasma-cancer treatment and in plasma-agriculture applications.

Here, we investigated the impact of a combination of lipid nitration and oxidation (nitro-oxidation) on the phospholipid membrane and its permeability by translocating RONS across the membrane. For this purpose, we applied molecular dynamics (MD) simulations, together with umbrella sampling (US) to reveal the permeation of RONS through various nitro-oxidized damaged membranes.

## 2. Simulation Setup

**Figure 1:**
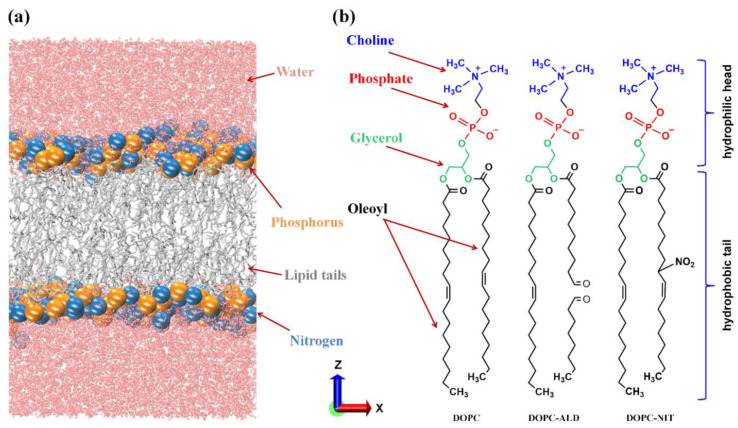
(**a**) Native DOPC PLB, with P and N atoms illustrated as larger beads for clarity. (**b**) Schematic depiction of the DOPC lipid molecule in its native, oxidized (DOPC-ALD), and nitrated (DOPC-NIT) states.

We conducted MD simulations to investigate the translocation of RONS, namely hydroxyl radical (OH), hydroperoxyl radical (HO_2_), hydrogen peroxide (H_2_O_2_), ozone (O_3_), nitric oxide (NO), nitrogen dioxide (NO_2_), dinitrogen tetroxide (N_2_O_4_), nitric acid (HNO_3_), *s-cis-*nitrous acid (*s-cis-*HONO), and *s-trans-*nitrous acid (*s-trans-*HONO), across both native and nitro-oxidized membranes. As a model system, we used the phospholipid bilayer (PLB), which served as a representation of the cell membrane. The area per lipid in simulations can vary depending on the conditions, and it has been reported to range from 65 to 70 Å^2^ in various studies [40,41]. These model systems typically include 40 to 65 water molecules per lipid, to ensure proper hydration. The experimentally measured area per lipid for DOPC was 67.4 ± 1.0 Å^2^ [42], which falls within the range of theoretical calculations. In our model system, we included 62.5 water molecules per lipid, and the calculated area per lipid was found to be 65 Å^2^ (cf. below Table 1 and Section 3), which is consistent with the experimental results. Our PLB consisted of 200 phospholipids (PLs) that were equally distributed in both layers of the membrane, in addition to 12,500 water molecules surrounding them from above and below (see Figure 1a). It is important to note that we used a higher number of water molecules per lipid in our model system, to facilitate umbrella sampling simulations. Moreover, inserting six of one of the RONS (e.g., 6 OH radicals) along the *z*-axis in our system required a larger simulation box. This was necessary to prevent interactions with periodic images. As a result, we included more water molecules, to ensure an appropriate distance between the RONS and the periodic boundary conditions. We considered native PLB consisting of 1,2-dioleoyl-sn-glycero-3-phosphocholine (DOPC) lipid molecules, as well as treated PLB consisting of both oxidized and nitrated forms of DOPC (see Figure 1b). As is clear from figure, the oxidation of DOPC led to the truncation of its fatty acid chain and the formation of aldehyde groups, while the nitration of DOPC led to the formation of a nitro group (-NO_2_) on one of its fatty acid tails [43]. The modifications possess three variations of the treated PLB, each of which differed in its composition (see Table 1). We selected the above (native and modified) PLs based on the following reasons: (i) DOPC lipid is a fundamental constituent of the plasma membrane in both the extracellular and cytoplasmic leaflets [44], (ii) aldehyde-oxidized lipid (DOPC-ALD) is one of the frequently observed oxidation products [45,46] and the main one responsible for increasing membrane permeability [47,48,49], and (iii) nitro PL (DOPC-NIT) is one of the main nitrated lipids observed during PL nitration [34,35]. It should be noted that the cellular membrane is composed of a variety of membrane constituents, such as PLs, proteins, sterols, and so on. In practice, the computational resources required for simulating the realistic membrane composition are quite expensive, since even the most elementary plasma membrane of a red blood cell is composed of more than 150 lipid species [50]. In this respect, we considered the principal lipid constituents in our simulations. Hence, in our model system, we chose DOPC as the primary component of the PLB. This particular lipid molecule is ubiquitous, being present in the endoplasmic reticulum, mitochondrion membrane, and liver cell plasma membrane at levels of approximately 40%, 44%, and 24%, respectively [50]. The initial configurations of the native and modified PLBs were generated using the Packmol software package [51]. All simulations were carried out using the GPU accelerated version of GROMACS-2021.4 software package [52,53]. We employed the GROMOS (53A6) united atom force field to simulate the behavior of native PLs [54]. To account for the presence of the aldehyde and nitro functional groups, we used the force field parameters developed for oxi- and nitro-lipids [35,55]. Additionally, we obtained the GROMOS-type force field parameters for the RONS investigated in this study from [56,57,58]. The choice of these parameters was based on their demonstrated ability to produce accurate water-to-alkane partition coefficients for ROS and RNS, as these coefficients are crucial for accurate modeling of transmembrane permeation. The force field parameters we utilized were further complemented by the parameters for NO particles that we had previously developed using the same methodologies as described in [56,57].

The model membranes were initially minimized employing the steepest descent algorithm prior to a 100 ps equilibration in the isothermal-isobaric (NPT) ensemble at 1 bar and 310 K. These equilibration runs utilized a semi-isotropic Parrinello–Rahman barostat with a compressibility and coupling constant of 4.5 × 10^−5^ bar^−1^ and 5.0 ps, respectively, along with a Nose–Hoover thermostat with a coupling constant of 0.5 ps. A cut-off radius of 1.0 nm was used for non-bonded (Lennard–Jones) and electrostatic (Coulomb) interactions. The particle mesh Ewald method was employed to handle electrostatic interactions, with a real space cut-off of 1.0 nm, coupled with a 0.16 nm spaced-grid for the reciprocal-space interactions. All simulations were accomplished using a time step of 2 fs, and periodic boundary conditions were implemented in all three dimensions. Furthermore, the production run was performed for 500 ns which was a sufficiently long to achieve an equilibrated state for our four model membranes.

**Figure 2:**
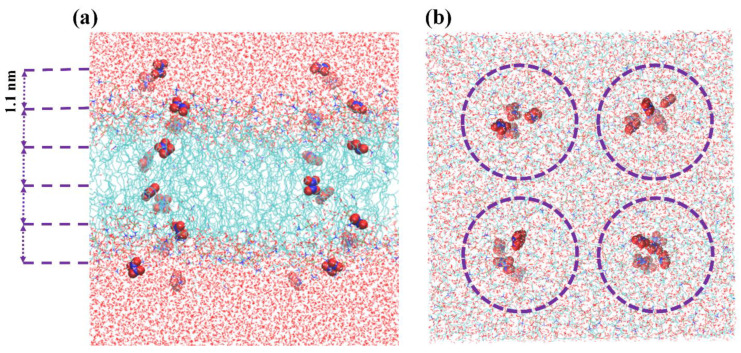
(**a**) Side and (**b**) top view of the PLB model system used in US simulations. The particles inserted into PLB were separated by 1.1 nm along the *z*-axis (see dashed lines). They were also quite far apart (i.e., within 4 nm) in the xy-plane (see dashed circles).

**Figure A1:**
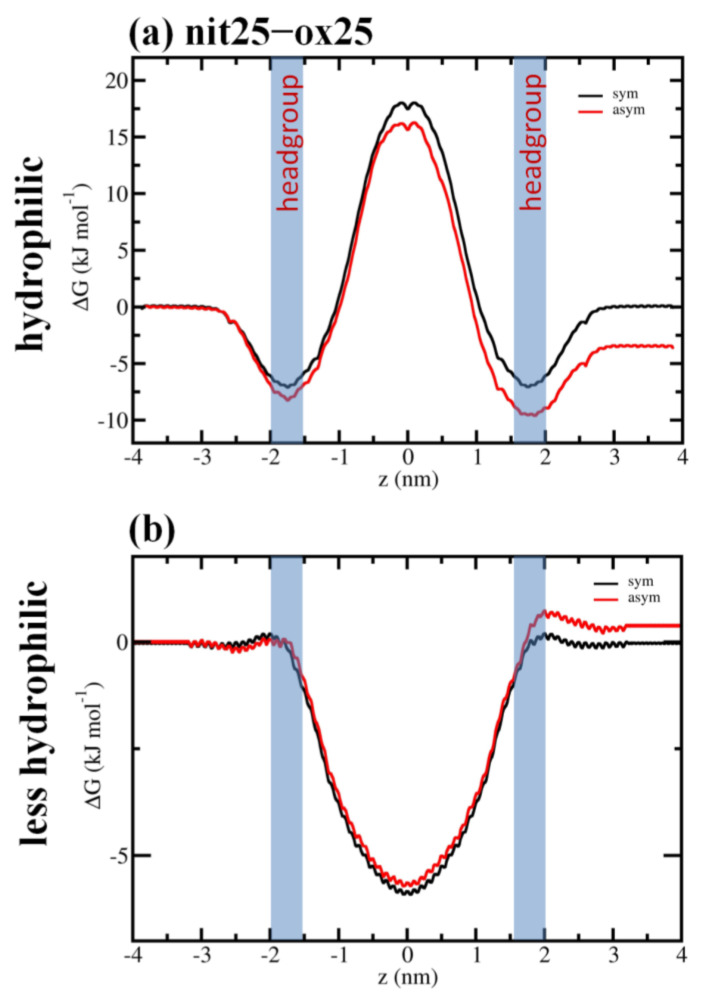
Comparison between the symmetric (i.e., symmetric to the center of the PLB) and full (asymmetric) FEPs of H_2_O_2_ (**a**) and NO (**b**) across the nit25–ox25 system.

To compute the free energy profiles (FEPs) associated with the translocation of ROS and RNS across the membranes, we utilized the US method [59,60]. The initial membrane structures for US simulations were chosen from the final 30 ns of the equilibration trajectory (of the production run) by extracting frames with 10 ns intervals (i.e., 470, 480, 490, and 500 ns). Thus, four replicas were used in our US simulations. In order to efficiently use the computational resources, the US simulations were carried out as follows: as mentioned above, four membrane structures were selected from the last 30 ns and, in each of the US simulations, six permeants were positioned along the membrane normal, while maintaining a distance of 1.1 nm between them (see Figure 2a). Moreover, to gain more statistics, we inserted 18 more permeants of the above species, varying their position in the xy-plane. These species were located sufficiently far from each other, i.e., beyond the cut-off radius, and they were allowed to move in the xy-plane, within the range of the applied flat-bottomed position restraint with a force constants of 500 kJ·mol^−1^ nm^−2^ and a radius of 0.5 nm (see Figure 2b). To clarify, the inserted species in our simulations did not interact with each other, as they were located beyond the specified cut-off radii. Despite traveling along the edge of the flat bottom restrained potential, the minimum distance between them in the xy-plane was approximately 3 nm (see Figure 2b). This ensured that the species remained sufficiently separated and prevented any unintended interactions during the simulation. To maintain the positions of permeants relative to the membrane center, their center-of-mass motion was also restrained along the *z*-axis using the harmonic bias with a force constant of 2000 kJ·mol^−1^ nm^−2^. The permeants were allowed to move freely in the xy-plane. Each system underwent a US simulation at NPT, consisting of 2 ns of equilibration followed by 4 ns of sampling (i.e., a total of 6 ns US simulation). The histograms obtained from the US simulations were collected and analyzed using the weighted histogram analysis method to calculate the associated FEP for each system [61]. We conducted eleven individual US simulations, in order to construct a single FEP. As a result, (6 × 11) × 4 = 264 US data points were collected along the membrane normal, each separated by 1.1 Å, for four FEP (i.e., 66 for each FEP). Thus, we obtained 16 individual FEPs (i.e., 4 permeants in each US run × 4 replicas) for each RONS (i.e., OH, HO_2_, H_2_O_2_, O_3_, NO, NO_2_, N_2_O_4_ HNO_3_, *s-cis-*HONO, and *s-trans-*HONO). In this way, the final FEP of each particle was obtained by averaging 16 individual FEPs. Since we used ten RONS and four PLB systems (see Table 1), a total of 11 US runs × 10 RONS × 4 PLB structures × 4 replicas = 1760 US simulations were performed, each lasting for 6 ns. Hence, a total of 10.56 µs of US simulations were carried out in this study. It is important to note that the data obtained for the FEP calculation heavily relied on the lipid composition surrounding the randomly placed RONS in the PLB. In order to obtain accurate free energy profiles (and corresponding barriers), a good statistical analysis is required. Additionally, to evaluate the convergence of the free energy results, it was necessary to examine the symmetry of the FEPs of each RONS to the center of the PLB. Our simulation results revealed that the full FEPs obtained in this study were very similar to the symmetrical ones, indicating the convergence of the simulations (see Appendix A, Figure A1 and cf the FEPs of HO_2_ (hydrophilic) and NO (less hydrophilic) across the nit25–ox25 system). It is worth noting that in reality, chemical reactions involving RONS may occur in the PLB. However, conventional non-reactive MD simulations cannot fully capture these processes, due to the limitations of the potential used. The electronic degrees of freedom required to describe chemical reactions are not explicitly taken into account in classical MD simulations. Owing to these limitations, we simulated membranes after oxidation/nitration. Nevertheless, US simulations can provide valuable information on the RONS permeation rate across the PLB before and after modification, as well as the most probable accumulation regions for RONS. These results allow for the study of position-dependent specific interactions in the membrane, which may have implications for biomedical applications.

## 3. Results and Discussion

**Figure 3:**
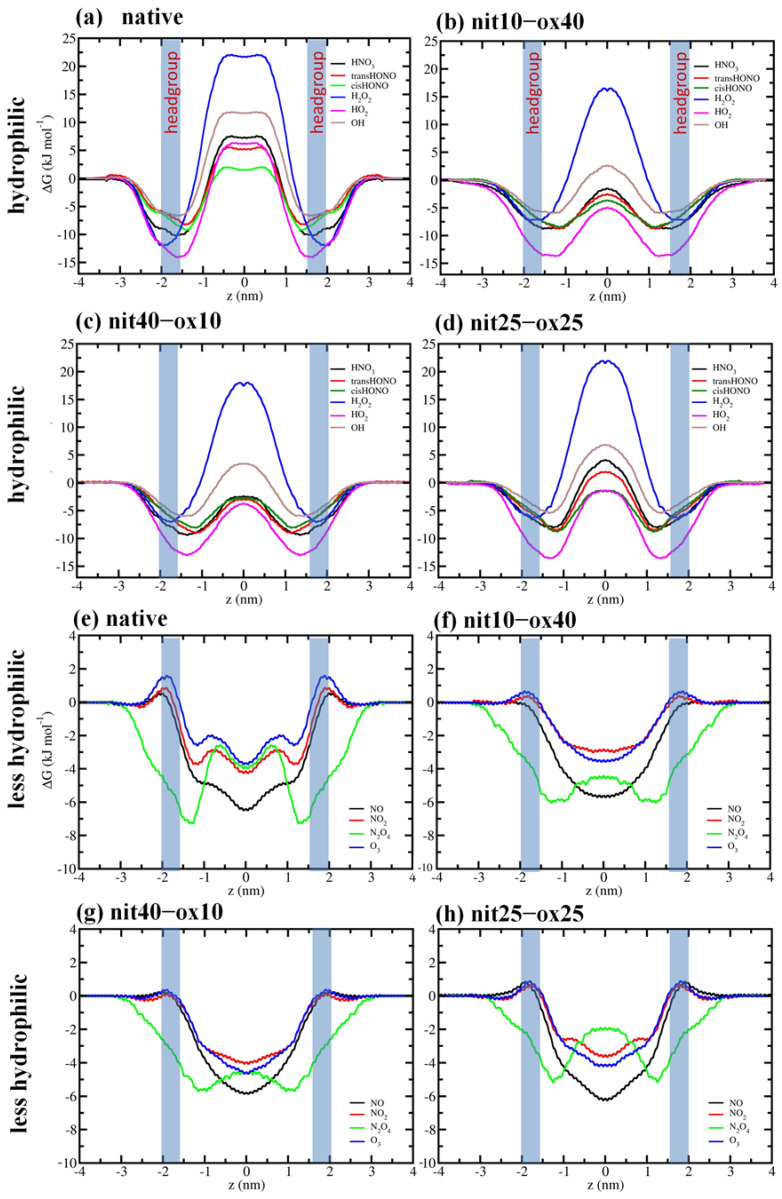
FEPs of the hydrophilic (**a**–**d**) and hydrophobic (**e**–**h**) RONS, across the native and modified PLBs.

In this study, our goal was to investigate the mechanisms of the permeation of RONS, i.e., OH, HO_2_, H_2_O_2_, O_3_, NO, NO_2_, N_2_O_4_, HNO_3_, *s-cis-*HONO, and *s-trans-*HONO through native and modified PLBs. Figure 3 shows the FEPs of RONS across both native and modified PLBs. 

The associated free energy barriers (ΔG) are given in Table 2.

In reference to hydrophilic species, each energy barrier was quantified using the difference of the minimum and maximum of the FEPs. Conversely, for the case of less hydrophilic species [62,63,64], the free energy barrier was observed within the headgroup region of PLB. When the hydrophilic RONS (i.e., HNO_3_, *s-cis-*HONO, *s-trans-*HONO, H_2_O_2_, HO_2_, and OH) are transferred from the aqueous phase to the interior of the membrane, the FEP initially decreases, attaining a minimum at the headgroup region, and subsequently rises as the RONS are become positioned deeper into the native PLB (see Figure 3a). As is clear, the free energy barrier was obtained at the core of the PLB in all cases. Adsorption at the headgroup region was considerably more robust for H_2_O_2_ and HO_2_, with H_2_O_2_ displaying the highest permeation barrier among all hydrophilic RONS. A previous simulation study found specific interactions between ROS and the membrane, which could be responsible for these trends [56]. It has been postulated that the adsorption of RONS onto the surface of PLBs is principally driven by two factors: (i) hydrogen bonding interactions between RONS and the carbonyl ester groups of the PL, and (ii) dispersion interactions with the headgroup region. Although all the hydrophilic RONS investigated in this study are capable of acting as hydrogen bond donors, H_2_O_2_ and HO_2_ possess an additional oxygen atom, which results in stronger dispersion interactions in comparison to the smaller OH radical. This may explain the differences in the adsorption tendencies of the hydrophilic RONS. Additionally, it is known that H_2_O_2_ has the ability to form twice as many hydrogen bonds in water as compared to OH and HO_2_ [56]. This may elucidate the reason behind the substantial permeation barrier observed for H_2_O_2_. It is noteworthy how fundamental principles of chemical bonding can be employed to elucidate distinctions in the comportment of *s-cis*-*s-trans* isomeric forms of nitrous acid. In the case of *s-trans*-HNO_2_, the alignment of discrete bond dipoles occurs in a harmonious manner, fostering a propitious interplay among them. Such felicitously aligned bond dipoles leads to the formation of a discernible molecular dipole, ultimately resulting in favorable hydration. On the contrary, the *s-cis* conformation gives rise to an adverse alignment of bond dipoles, rendering it less polar when juxtaposed with its *s-trans* counterpart. Our straightforward qualitative explanation coheres with experimentally determined dipole moments: 1.85 D [65] and 1.42 D [66] for *s-trans* and *s-cis* isomers, respectively. Furthermore, the dissimilarities in the chemical structures of these isomers also contributed to the contrasting topological polar surfaces exhibited by them, with the *s-trans* form boasting a larger polar surface in comparison to the *s-cis* form. These electronic and structural disparities were evinced in the ∆G of these isomers: the *s-trans* isomer underwent more favorable hydration and exhibited a larger ∆G in the headgroup region, while the *s-cis* isomer experienced less favorable hydration and confronted a diminished ∆G barrier in the headgroup region (see Table 2 and Figure 3a–d). In the event that the PLB underwent nitro-oxidation, the permeation pattern exhibited similar qualitative tendencies, albeit with comparatively less free energy barriers (see Figure 3b–d). The oxidation of PL produced functional groups and fragments that enhanced the hydrophilicity of the membrane core, thereby increasing the permeability of the PLB to RONS [67]. Whilst the permeation barrier remained comparatively high for H_2_O_2_, it became significantly reduced for OH in all cases of nitro-oxidized PLB. Nevertheless, the nitro-oxidation of the PLB reduced the translocation free energy barriers of all hydrophilic species compared to native PLB. Notably, with regards to HO_2_, nitro-oxidation of the PLB apparently induced a modification in its partition behavior. The FEPs showed that HO_2_ may manifest a preference for the PLB core, as nitro-oxidation occurs. This is a particularly important effect given that HO_2_ is the protonated form of the biologically significant superoxide radical ($O_{2}^{-}$). These radicals play a pivotal role in biological systems, acting as essential mediators in redox signaling pathways. Their controlled production and regulation are crucial for maintaining cellular homeostasis, as they participate in various physiological processes, such as cell signaling, immune response modulation, and regulation of vascular tone. Nevertheless, we would like to emphasize that this conclusion is still hypothetical. The HO_2_ model was initially parametrized to replicate its experimentally determined hydration free energy [56]. The consideration of less hydrophilic solvation was precluded, due to a lack of available experimental data. To draw a more definitive conclusion, it would be necessary to assess the HO_2_ model’s ability to describe its solvation free energy in non-polar solvents.

Overall, the less hydrophilic RONS exhibit substantially lower permeation barriers than their hydrophilic counterparts (see Figure 3e–h). They do not participate in hydrogen bonding interactions in the aqueous layer, which facilitates their diffusion from the aqueous phase to the core of the PLB. The maximum energy of all less hydrophilic RONS is located near the water–lipid interface, specifically in the headgroup region, which is mainly attributed to the polarity of the headgroup region. The values of maximum energy for all less hydrophilic RONS in all nitro-oxidized PLB systems are similar, having a slightly higher barrier in the case of the native PLB (Figure 3e–h). Additionally, the FEPs of the less hydrophilic RONS exhibit their minima at the center of the PLB, indicating the preference of these species (especially, NO, NO_2_, and O_3_) to accumulate in the PLB center. The FEPs imply that NO, being virtually nonpolar, has a high tendency to accumulate inside the PLB, and triatomic species such as NO_2_ and O_3_ also represents similar FEPs, which conserve a residual dipole moment [57]. N_2_O_4_ exhibits a behavior distinctive from other hydrophilic species, as it does not encounter any energy barrier in the headgroup region; rather, a minimum free energy is observed in close proximity to this area. When it penetrates deeper into the PLB core, the free energy gradually rises, but it is still lower than that in bulk water. The barrier near the center of the native PLB (i.e., at a distance of about 0.7 nm) is caused by a double bond present in the oleoyl tails, which makes the lipid chains bent. This barrier also represents similar changes for the other less hydrophilic species (see NO_2_ and O_3_ in Figure 3e). These findings imply that N_2_O_4_ has strong interaction with the membrane surface but partitions almost equally between the aqueous phase and the PLB core. The FEP of N_2_O_4_ exhibits characteristics of both hydrophilic and less hydrophilic species. The geometry of N_2_O_4_ is such that it has no dipole moment, but the partial charges of N and O atoms are relatively significant (+0.584e and −0.292e, respectively) [57]. It is possible for N_2_O_4_ to exhibit a strong quadrupole moment, which may explain its tendency to accumulate around the polar headgroup region, despite being less hydrated in this area compared to the aqueous phase. Despite undergoing nitro-oxidation, the permeation FEPs of less hydrophilic RONS remain largely unaffected, except for their smoothing and the near disappearance of the barrier near the headgroup (see Figure 3e–h). The local free energy barriers present at approximately |0.7| nm also disappears (see Figure 3f–h). This phenomenon can be attributed to the cleavage of the lipid tails (in the case of oxidation) and the consequent formation of aldehyde groups (see Figure 1b). The elimination of the localized free energy barriers can be attributed to the fluidity of the membrane. This fluidity is caused by an increase in the surface area of the PLB (area per lipid) and a decrease in the membrane thickness due to disordered lipid tails (see Table 3) [67]. Nevertheless, less hydrophilic species, except for N_2_O_4_, still tend to accumulate at the center of the PLB.

The findings from our simulations are in accordance with previous experimental studies on PLB permeability. Specifically, our simulation results demonstrate that less hydrophilic species such as NO exhibit a permeability that is 3–6 orders of magnitude higher than that of hydrophilic RONS such as H_2_O_2_ [68,69]. Thus, the incorporation of membrane-embedded aquaporin channels or pores induced by powerful electric fields [67] becomes crucial, to enhance the transport of hydrophilic RONS into and out of the cell. In fact, our recent findings indicate that the permeability of H_2_O_2_ through AQP is two orders of magnitude greater than through the PLB [32]. Indeed, for less hydrophilic RONS, transmembrane permeation can occur, even without the presence of channels and pores. The literature has also demonstrated this, where a higher permeability coefficient was reported [70], ranging from 18 to 93 cm s^−1^ for NO [69,71,72], 5 cm s^−1^ for NO_2_ [72], and 12 to 157 cm s^−1^ for O_2_ [69,71,73,74,75], whereas for H_2_O_2_, the permeability coefficient varies between 4 × 10^−4^ and 1.2 × 10^−2^ cm s^−1^ [70].

As previously discussed, the cell membrane is inherently intricate and composed of various membrane constituents. As a result, these constituents may also impact the penetration of RONS into the cytoplasm. It is evident that examining the impact of all these constituents is unfeasible. In a previous investigation, the impact of cholesterol on the FEPs of ROS (i.e., OH, HO, H_2_O_2_ and O_2_) was explored [76]. According to this study, the presence of cholesterol raised the lipid order, leading to an increase in the free energy barrier for ROS penetration. Thus, the membrane system which included cholesterol may influence the FEPs of the studied RONS, by raising their free energy barriers.

## 4. Conclusions

This research is of significant importance for the field of plasma medicine and marks a significant stride towards comprehending the diverse translocation abilities of various RONS (produced by CAP) across the native and nitro-oxidized cell membrane.

Overall, we may infer that less hydrophilic species, namely NO, NO_2_, N_2_O_4_, and O_3_, exhibit a higher penetration ability across both native and nitro-oxidized PLBs, in comparison to their hydrophilic counterparts, such as HNO_3_, *s-cis-*HONO, *s-trans-*HONO, H_2_O_2_, HO_2_, and OH. The nitro-oxidation of the PLB induced by processes such as CAP does not have a significant impact on the free energy barriers of less hydrophilic species. As is clear, the free energy barrier is slightly higher when there is 25% nitrated lipids + 25% oxidized lipids (especially for HNO_3_, *s-trans-*HONO, and HO_2_). This result is in agreement with the previous results of the study, where a mixture between 50% oxidized lipids and 50% nitrated lipids presented a higher free energy barrier for water permeation than 100% nitrated membranes [38]. However, this reduces the barriers of hydrophilic RONS, thereby increasing their probability of translocation across the nitro-oxidized PLB.

These computational studies shed light on the mechanisms underlying the action of RONS on native and modified membranes and provide valuable information about processes at the molecular level. Further detailed analyses are necessary, to unravel the synergistic function of these species in intracellular signaling pathways. This, in turn, will help in better understanding the behavior of biological systems, as well as provide a basis for future research and the development of new treatment options and targeted technologies.

## Figures and Tables

**Table 1:** The composition of the PLB used in our MD simulations. Here, the abbreviations used for nitrated and oxidized bilayers are nit and ox, respectively, and the numbers given with them indicate their molar fractions.

PLB Type	DOPC	Nitrated DOPC	Oxidized DOPC
native	100%	-	-
nit10-ox40	50%	10%	40%
nit40-ox10	50%	40%	10%
nit25-ox25	50%	25%	25%

**Table 2:** The permeation free energy barriers (ΔG) for hydrophilic RONS from the head group to the center of the PLB. The less hydrophilic RONS permeation barrier from water into the head group of PLB.

	ΔG for Hydrophilic RONS (kJ·mol^−1^)			
	Native	nit10–ox40	nit40–ox10	nit25–ox25
**HNO_3_**	17.5 ± 2	7.7 ± 2.1	7.3 ± 1.2	12.2 ± 1.1
***s-trans-*HONO**	13.9 ± 2	6.1 ± 2.2	6.1 ± 1.2	10.6 ± 1.2
***s-cis-*HONO**	11.3 ± 3	5.1 ± 2.1	5.6 ± 1.2	7.9 ± 1.3
**H_2_O_2_**	34.0 ± 3	23.5 ± 2.1	25.7 ± 1.3	28.3 ± 1.1
**HO_2_**	20.0 ± 3	8.8 ± 2.1	9.5 ± 1.1	12.5 ± 1.2
**OH**	18.5 ± 3	3.7 ± 2.3	9.4 ± 1.1	12.4 ± 1.3
	**ΔG for Less Hydrophilic RONS (kJ·mol^−1^)**			
	**Native**	**nit10**–**ox40**	**nit40**–**ox10**	**nit25**–**ox25**
**NO**	0.5	0.1	0.3	1.0
**NO_2_**	0.9	0.4	0.1	0.6
**N_2_O_4_**	4.7	1.7	1.2	3.4
**O_3_**	1.6	0.8	0.5	1.0

**Table 3:** The types of PLBs and corresponding area per lipids (APLs) calculated from the last 100 ns of the simulation trajectory.

System	APL (nm^2^)
native	0.6507 ± 0.0001
nit10–ox40	0.7651 ± 0.0001
nit20–ox30	0.7448 ± 0.0002
nit25–ox25	0.7345 ± 0.0003
nit30–ox20	0.7338 ± 0.0001
nit40–ox10	0.7216 ± 0.0002

## Data Availability

All the data are included in the main text.

## References

[1] Bárdos L., Baránková H. (2010). Cold atmospheric plasma: Sources, processes, and applications. Thin Solid Film..

[2] Benedikt J., Hefny M.M., Shaw A., Buckley B., Iza F., Schäkermann S., Bandow J. (2018). The fate of plasma-generated oxygen atoms in aqueous solutions: Non-equilibrium atmospheric pressure plasmas as an efficient source of atomic O (aq). Phys. Chem. Chem. Phys..

[3] Uchiyama H., Ishikawa K., Zhao Q.-L., Andocs G., Nojima N., Takeda K., Krishna M.C., Ishijima T., Matsuya Y., Hori M. (2018). Free radical generation by non-equilibrium atmospheric pressure plasma in alcohol–water mixtures: An EPR-spin trapping study. J. Phys. D Appl. Phys..

[4] Weltmann K.D., Polak M., Masur K., von Woedtke T., Winter J., Reuter S. (2012). Plasma processes and plasma sources in medicine. Contrib. Plasma Phys..

[5] Georgescu N., Lupu A.R. (2010). Tumoral and normal cells treatment with high-voltage pulsed cold atmospheric plasma jets. IEEE Trans. Plasma Sci..

[6] Fridman G., Friedman G., Gutsol A., Shekhter A.B., Vasilets V.N., Fridman A. (2008). Applied plasma medicine. Plasma Process. Polym..

[7] Domonkos M., Tichá P., Trejbal J., Demo P. (2021). Applications of cold atmospheric pressure plasma technology in medicine, agriculture and food industry. Appl. Sci..

[8] Von Woedtke T., Reuter S., Masur K., Weltmann K.-D. (2013). Plasmas for medicine. Phys. Rep..

[9] Isbary G., Morfill G., Schmidt H., Georgi M., Ramrath K., Heinlin J., Karrer S., Landthaler M., Shimizu T., Steffes B. (2010). A first prospective randomized controlled trial to decrease bacterial load using cold atmospheric argon plasma on chronic wounds in patients. Br. J. Dermatol..

[10] Penkov O.V., Khadem M., Lim W.-S., Kim D.-E. (2015). A review of recent applications of atmospheric pressure plasma jets for materials processing. J. Coat. Technol. Res..

[11] Assadi I., Guesmi A., Baaloudj O., Zeghioud H., Elfalleh W., Benhammadi N., Khezami L., Assadi A.A. (2021). Review on inactivation of airborne viruses using non-thermal plasma technologies: From MS2 to coronavirus. Environ. Sci. Pollut. Res. Int..

[12] Tanaka H., Mizuno M., Ishikawa K., Toyokuni S., Kajiyama H., Kikkawa F., Hori M. (2018). Molecular mechanisms of non-thermal plasma-induced effects in cancer cells. Biol. Chem..

[13] Joshi S.G., Paff M., Friedman G., Fridman G., Fridman A., Brooks A.D. (2010). Control of methicillin-resistant Staphylococcus aureus in planktonic form and biofilms: A biocidal efficacy study of nonthermal dielectric-barrier discharge plasma. Am. J. Infect. Control.

[14] Chen Z., Chen G., Obenchain R., Zhang R., Bai F., Fang T., Wang H., Lu Y., Wirz R.E., Gu Z. (2022). Cold atmospheric plasma delivery for biomedical applications. Mater. Today.

[15] Misra N., Bhatt S., Arefi-Khonsari F., Kumar V. (2021). State of the art in nonthermal plasma processing for biomedical applications: Can it help fight viral pandemics like COVID-19?. Plasma Process. Polym..

[16] Kong M.G., Kroesen G., Morfill G., Nosenko T., Shimizu T., Van Dijk J., Zimmermann J. (2009). Plasma medicine: An introductory review. New J. Phys..

[17] Keidar M., Walk R., Shashurin A., Srinivasan P., Sandler A., Dasgupta S., Ravi R., Guerrero-Preston R., Trink B. (2011). Cold plasma selectivity and the possibility of a paradigm shift in cancer therapy. Br. J. Cancer.

[18] González-Mendoza B., López-Callejas R., Rodríguez-Méndez B.G., Eguiluz R.P., Mercado-Cabrera A., Valencia-Alvarado R., Betancourt-Ángeles M., de Lourdes Reyes-Frías M., Reboyo-Barrios D., Chávez-Aguilar E. (2019). Healing of wounds in lower extremities employing a non-thermal plasma. Clin. Plasma Med..

[19] Lee J.-S., Shin H.-S., Seok J.-W., Jang G.-W., Beag Y.-H. (2009). Surface Modification of Polystyrene (PS) by Atmospheric Pressure Plasma. J. Korean Vac. Soc..

[20] Razzokov J., Fazliev S., Kodirov A., AttrI P., Chen Z., Shiratani M. (2022). Mechanistic insight into permeation of plasma-generated species from vacuum into water bulk. Int. J. Mol. Sci..

[21] Van der Paal J., Hong S.-H., Yusupov M., Gaur N., Oh J.-S., Short R.D., Szili E.J., Bogaerts A. (2019). How membrane lipids influence plasma delivery of reactive oxygen species into cells and subsequent DNA damage: An experimental and computational study. Phys. Chem. Chem. Phys..

[22] Kaushik N., Kaushik N.K., Kim C.H., Choi E.H. (2014). Oxidative stress and cell death induced in U-937 human monocytic cancer cell line by non-thermal atmospheric air plasma soft jet. Sci. Adv. Mater..

[23] Xia W., Budge S.M. (2017). Techniques for the analysis of minor lipid oxidation products derived from triacylglycerols: Epoxides, alcohols, and ketones. Compr. Rev. Food Sci. Food Saf..

[24] Volinsky R., Cwiklik L., Jurkiewicz P., Hof M., Jungwirth P., Kinnunen P.K. (2011). Oxidized phosphatidylcholines facilitate phospholipid flip-flop in liposomes. Biophys. J..

[25] Elbaradei A., Wang Z., Malmstadt N.J.L. (2022). Oxidation of Membrane Lipids Alters the Activity of the Human Serotonin 1A Receptor. Langmuir.

[26] Corvalan N.A., Caviglia A.F., Felsztyna I., Itri R., Lascano R.J.L. (2020). Lipid hydroperoxidation effect on the dynamical evolution of the conductance process in bilayer lipid membranes: A condition toward criticality. Langmuir.

[27] Ouchi Y., Unoura K., Nabika H. (2019). Role of oxidized lipids in permeation of H_2_O_2_ through a lipid membrane: Molecular mechanism of an inhibitor to promoter switch. Sci. Rep..

[28] De Rosa R., Spinozzi F., Itri R. (2018). Hydroperoxide and carboxyl groups preferential location in oxidized biomembranes experimentally determined by small angle X-ray scattering: Implications in membrane structure. Biochim. Biophys. Acta (BBA)-Biomembr..

[29] Neto A.J., Cordeiro R.M. (2016). Molecular simulations of the effects of phospholipid and cholesterol peroxidation on lipid membrane properties. Biochim. Biophys. Acta (BBA)-Biomembr..

[30] Singer S.J., Nicolson G.L. (1972). The Fluid Mosaic Model of the Structure of Cell Membranes: Cell membranes are viewed as two-dimensional solutions of oriented globular proteins and lipids. Science.

[31] Razzokov J., Yusupov M., Vanuytsel S., Neyts E.C., Bogaerts A. (2017). Phosphatidylserine flip-flop induced by oxidation of the plasma membrane: A better insight by atomic scale modeling. Plasma Process. Polym..

[32] Yusupov M., Razzokov J., Cordeiro R.M., Bogaerts A. (2019). Transport of reactive oxygen and nitrogen species across aquaporin: A molecular level picture. Oxidative Med. Cell. Longev..

[33] Oliveira M.C., Yusupov M., Bogaerts A., Cordeiro R.M. (2022). Biophysics. Distribution of lipid aldehydes in phase-separated membranes: A molecular dynamics study. Arch. Biochem. Biophys..

[34] Melo T., Domingues P., Ferreira R., Milic I., Fedorova M., Santos S.M., Segundo M.A., Domingues M.R.M. (2016). Recent Advances on Mass Spectrometry Analysis of Nitrated Phospholipids. Anal. Chem..

[35] Oliveira M.C., Yusupov M., Bogaerts A., Cordeiro R.M. (2020). How do nitrated lipids affect the properties of phospholipid membranes?. Arch. Biochem. Biophys..

[36] Razzokov J., Yusupov M., Cordeiro R.M., Bogaerts A. (2018). Atomic scale understanding of the permeation of plasma species across native and oxidized membranes. J. Phys. D Appl. Phys..

[37] Nasri Z., Ahmadi M., Striesow J., Ravandeh M., von Woedtke T., Wende K.J.I. (2022). Insight into the Impact of Oxidative Stress on the Barrier Properties of Lipid Bilayer Models. Int. J. Mol. Sci..

[38] Oliveira M.C., Yusupov M., Cordeiro R.M., Bogaerts A. (2021). Unraveling the permeation of reactive species across nitrated membranes by computer simulations. Comput. Biol. Med..

[39] Privat-Maldonado A., Bengtson C., Razzokov J., Smits E., Bogaerts A. (2019). Modifying the tumour microenvironment: Challenges and future perspectives for anticancer plasma treatments. Cancers.

[40] Klauda J.B., Venable R.M., Freites J.A., O’Connor J.W., Tobias D.J., Mondragon-Ramirez C., Vorobyov I., MacKerell A.D., Pastor R.W. (2010). Update of the CHARMM all-atom additive force field for lipids: Validation on six lipid types. J. Phys. Chem. B.

[41] Kučerka N., Tristram-Nagle S., Nagle J.F. (2006). Structure of fully hydrated fluid phase lipid bilayers with monounsaturated chains. J. Membr. Biol..

[42] Kučerka N., Nagle J.F., Sachs J.N., Feller S.E., Pencer J., Jackson A., Katsaras J. (2008). Lipid bilayer structure determined by the simultaneous analysis of neutron and X-ray scattering data. Biophys. J..

[43] Schopfer F., Batthyany C., Baker P., Bonacci G., Cole M., Rudolph V., Groeger A., Rudolph T., Nadtochiy S., Brookes P. (2009). Detection and quantification of protein adduction by electrophilic fatty acids: Mitochondrial generation of fatty acid nitroalkene derivatives. Free Radic. Biol. Med..

[44] Matosevic S., Paegel B.M. (2013). Layer-by-layer cell membrane assembly. Nat. Chem..

[45] Davis S.E., Ide M.S., Davis R.J. (2013). Selective oxidation of alcohols and aldehydes over supported metal nanoparticles. Green Chem..

[46] Yusupov M., Wende K., Kupsch S., Neyts E.C., Reuter S., Bogaerts A. (2017). Effect of head group and lipid tail oxidation in the cell membrane revealed through integrated simulations and experiments. Sci. Rep..

[47] Runas K.A., Malmstadt N. (2015). Low levels of lipid oxidation radically increase the passive permeability of lipid bilayers. Soft Matter.

[48] Bacellar I.O., Oliveira M.C., Dantas L.S., Costa E.B., Junqueira H.C., Martins W.K., Durantini A.M., Cosa G., Di Mascio P., Wainwright M. (2018). Photosensitized membrane permeabilization requires contact-dependent reactions between photosensitizer and lipids. J. Am. Chem. Soc..

[49] Oliveira M.C., Yusupov M., Bogaerts A., Cordeiro R.M. (2021). Lipid oxidation: Role of membrane phase-separated domains. J. Chem. Inf. Model..

[50] Alberts B., Johnson A., Lewis J., Morgan D., Raff M., Roberts K., Walter P. (2014). Molecular Biology of the Cell.

[51] Martínez L., Andrade R., Birgin E.G., Martínez J.M. (2009). PACKMOL: A package for building initial configurations for molecular dynamics simulations. J. Comput. Chem..

[52] Van Der Spoel D., Lindahl E., Hess B., Groenhof G., Mark A.E., Berendsen H.J. (2005). GROMACS: Fast, flexible, and free. J. Comput. Chem..

[53] Kutzner C., Páll S., Fechner M., Esztermann A., de Groot B.L., Grubmüller H. (2019). More bang for your buck: Improved use of GPU nodes for GROMACS 2018. J. Comput. Chem..

[54] Oostenbrink C., Villa A., Mark A.E., Van Gunsteren W.F. (2004). A biomolecular force field based on the free enthalpy of hydration and solvation: The GROMOS force-field parameter sets 53A5 and 53A6. J. Comput. Chem..

[55] Wong-ekkabut J., Xu Z., Triampo W., Tang I.M., Peter Tieleman D., Monticelli L. (2007). Effect of Lipid Peroxidation on the Properties of Lipid Bilayers: A Molecular Dynamics Study. Biophys. J..

[56] Cordeiro R.M. (2014). Reactive oxygen species at phospholipid bilayers: Distribution, mobility and permeation. Biochim. Biophys. Acta (BBA)-Biomembr..

[57] Cordeiro R.M. (2018). Reactive oxygen and nitrogen species at phospholipid bilayers: Peroxynitrous acid and its homolysis products. J. Phys. Chem. B.

[58] Cordeiro R.M., Yusupov M., Razzokov J., Bogaerts A. (2020). Parametrization and molecular dynamics simulations of nitrogen oxyanions and oxyacids for applications in atmospheric and biomolecular sciences. J. Phys. Chem. B.

[59] Torrie G.M., Valleau J.P. (1977). Nonphysical sampling distributions in Monte Carlo free-energy estimation: Umbrella sampling. J. Comput. Phys..

[60] Kästner J. (2011). Umbrella sampling. Wiley Interdiscip. Rev. Comput. Mol. Sci..

[61] Kumar S., Rosenberg J.M., Bouzida D., Swendsen R.H., Kollman P.A. (1992). The weighted histogram analysis method for free-energy calculations on biomolecules. I. The method. J. Comput. Chem..

[62] Squadrito G.L., Postlethwait E.M. (2009). On the hydrophobicity of nitrogen dioxide: Could there be a “lens” effect for NO_2_ reaction kinetics?. Nitric Oxide.

[63] Hardy M., Zielonka J., Karoui H., Sikora A., Michalski R., Podsiadły R., Lopez M., Vasquez-Vivar J., Kalyanaraman B., Ouari O.J.A. (2018). Detection and characterization of reactive oxygen and nitrogen species in biological systems by monitoring species-specific products. Antioxid. Redox Signal..

[64] Goss S.P., Singh R.J., Hogg N., Kalyanaraman B. (1999). Reactions of· NO,· NO_2_ and peroxynitrite in membranes: Physiological implications. Free Radic. Res..

[65] Cox A.P., Brittain A.H., Finnigan D.J. (1971). Microwave spectrum, structure, dipole moment and quadrupole coupling constants of cis and trans nitrous acids. Trans. Faraday Soc..

[66] Cox A.P., Kuczkowski R.L. (1966). The Microwave Spectrum, Structure, Dipole Moment, and Quadrupole Coupling Constants of trans-Nitrous Acid1a. J. Am. Chem. Soc..

[67] Yusupov M., Van der Paal J., Neyts E.C., Bogaerts A. (2017). Synergistic effect of electric field and lipid oxidation on the permeability of cell membranes. Biochim. Biophys. Acta (BBA)-Gen. Subj..

[68] Möller M.N., Li Q., Lancaster J.R., Denicola A. (2007). Acceleration of nitric oxide autoxidation and nitrosation by membranes. IUBMB Life.

[69] Subczynski W.K., Lomnicka M., Hyde J.S. (1996). Permeability of nitric oxide through lipid bilayer membranes. Free Radic. Res..

[70] Möller M.N., Lancaster J.R., Denicola A. (2008). The interaction of reactive oxygen and nitrogen species with membranes. Curr. Top. Membr..

[71] Denicola A., Souza J.M., Radi R., Lissi E. (1996). Nitric oxide diffusion in membranes determined by fluorescence quenching. Arch. Biochem. Biophys..

[72] Signorelli S., Möller M.N., Coitiño E.L., Denicola A. (2011). Nitrogen dioxide solubility and permeation in lipid membranes. Arch. Biochem. Biophys..

[73] Subczynski W.K., Hopwood L.E., Hyde J.S. (1992). Is the mammalian cell plasma membrane a barrier to oxygen transport?. J. Gen. Physiol..

[74] Subczynski W.K., Hyde J.S., Kusumi A. (1989). Oxygen permeability of phosphatidylcholine--cholesterol membranes. Proc. Natl. Acad. Sci. USA.

[75] Widomska J., Raguz M., Subczynski W.K. (2007). Oxygen permeability of the lipid bilayer membrane made of calf lens lipids. Biochim. Biophys. Acta (BBA)-Biomembr..

[76] Van der Paal J., Verheyen C., Neyts E.C., Bogaerts A. (2017). Hampering effect of cholesterol on the permeation of reactive oxygen species through phospholipids bilayer: Possible explanation for plasma cancer selectivity. Sci. Rep..

